# A Comparative Analysis of Muscle Nutritional Composition, Texture, Microstructure, and Metabolomics: Hybrid Sturgeon (*Acipenser baerii* Brandt ♀ × *Acipenser schrenckii* Brandt ♂) Versus Its Parent Varieties

**DOI:** 10.3390/foods15101665

**Published:** 2026-05-10

**Authors:** Guanling Xu, Wei Xing, Ying Zhang, Tingting Song, Tieliang Li, Lin Luo, Huanhuan Yu

**Affiliations:** Fisheries Research Institute, Beijing Academy of Agriculture and Forestry Sciences, Beijing 100068, China; xuguanling@baafs.net.cn (G.X.); xingwei@baafs.net.cn (W.X.); zhangying@baafs.net.cn (Y.Z.); songtingting@baafs.net.cn (T.S.); litieliang@baafs.net.cn (T.L.); luolin@baafs.net.cn (L.L.)

**Keywords:** flesh quality, flavor, metabolite profiling

## Abstract

The hybrid sturgeon (HS, *Acipenser baerii* Brandt ♀ × *A*. *schrenckii* Brandt ♂) was compared with its parental varieties (Siberian sturgeon (SS) and Amur sturgeon (AS)) to evaluate muscle quality differences. Three sturgeon species were bred from the same batch, reared under identical conditions until three years of age, and then male fish from each species (AS, 3.2 ± 0.18 kg; SS, 2.5 ± 0.14 kg; HS, 3.5 ± 0.21 kg) were sampled for analysis of muscle nutritional composition, texture, microstructure, and metabolomics. Results showed no significant differences in proximate composition, hydrolyzed amino acids, pH, or water-holding capacity among the three groups. However, HS exhibited higher gumminess and chewiness than both parent species, as well as greater hardness and springiness compared with the SS. Muscle fiber density was higher in HS than in the AS, but no significant difference was observed between HS and SS. Levels of free amino acids (Val, Ile, Ala) were lower in HS than in AS. In terms of fatty acid profiles, HS showed elevated polyunsaturated fatty acids compared with SS, resembling the pattern observed in AS. Muscle color of HS was similar to that of SS, whereas its a* value differed from those of AS. Metabolomics identified differential metabolites (GABA, D-glucosaminic acid, AP4) enriched in pathways such as ABC transporters, protein digestion and absorption, and amino acid metabolism. Overall, HS combines improved texture traits with meat quality attributes resembling SS (muscle color, free amino acids) and AS (polyunsaturated fatty acids). These characteristics suggest that HS possesses a distinctive combination of meat quality traits.

## 1. Introduction

The high-value caviar and delicate flesh of sturgeons characterize these ancient members of the Acipenseridae family, distinguishing them in the global marketplace [1]. China, as the world’s major sturgeon supplier accounting for about 85% of global production, had reached an output of 149,376 tons by 2023, establishing sturgeon as an economically important species in its aquaculture sector [2,3]. The application of selective breeding and hybridization has been widely utilized in the sturgeon industry to enhance major commercial characteristics, including growth performance, disease resilience, and flesh quality [4]. A notable achievement is the cultivation of “Jinglong 1”. At present, the Siberian hybrid sturgeon, represented by the “Jinglong 1” variety, has become the dominant farmed species in China, owing to its superior growth performance and higher feed efficiency compared to its parent species [5]. Since muscle quality is a critical determinant of consumer acceptance and commercial viability in edible fish [6,7], it is equally worth asking whether the meat quality of the “Jinglong 1” hybrid sturgeon also possesses advantages comparable to its growth and disease resistance. However, no systematic study has yet compared the muscle quality of this hybrid with that of its parental species.

Multiple factors influence muscle quality, including nutritional composition, textural properties, microstructural characteristics, and metabolite profiles [7,8]. The nutritional composition of fish muscle, including protein content, lipid profile, amino acids, and fatty acids, directly determines its dietary value and health benefits [9]. Textural properties serve as critical determinants of sensory perception and market acceptance in filet, with their characteristics being fundamentally governed by microstructural features including myofiber density, collagen distribution patterns, and intramuscular adipocyte deposition [10]. Metabolomics, as a powerful analytical platform for characterizing small-molecule metabolites, represents the most phenotypically proximal omics approach that contributes to elucidating fundamental biochemical mechanisms governing muscle quality variations [11,12]. Recent studies on the muscle quality of Siberian hybrid sturgeon have primarily investigated the impacts of rearing environment [13] and dietary intervention [14,15], along with comparisons across different size groups or different body parts within the same size class [16,17]. Siberian sturgeon is known for its high intramuscular fat content [18], whereas Amur sturgeon exhibits larger myofiber diameter in muscle texture [1]. Whether the “Jinglong 1” hybrid inherits meat quality traits from both parents or exhibits a unique metabolic profile underlying its muscle characteristics remains unclear.

Accordingly, the primary objective of this study was to characterize the similarities and differences in meat quality between the hybrid sturgeon and its parent species. To this end, we performed a comprehensive comparative analysis of muscle nutritional composition, texture, microstructure, and metabolomic profiles. Specifically, we aimed to determine whether the hybrid inherits favorable traits from both parents or develops unique quality attributes, as assessed by phenotypic traits and metabolomic pathway analysis. The findings will provide a scientific basis for the commercial promotion of Siberian hybrid sturgeon represented by the “Jinglong 1” variety and contribute to sturgeon breeding strategies aimed at optimizing meat quality.

## 2. Materials and Methods

### 2.1. Animals and Samples Collection

AS, SS and HS were obtained from a commercial farm (Yanqing, Beijing, China). The three sturgeon species were bred from the same batch and maintained under identical conditions until three years of age. After one year of age, 60 sturgeons of each species are randomly selected and distributed into 4 repeated tanks (15 fish per tank). All tanks (diameter 3.0 m, water depth approximately 0.45 m) were maintained under the same environmental conditions in a recirculated rearing system: temperature 16.5 ± 1.0 °C, dissolved oxygen > 7.5 mg/L, pH 8.1 ± 0.2, and nitrite < 0.1 mg/L. Throughout the rearing period, fish were fed standardized commercial diets (Diet 1 before 6 months of age and Diet 2 thereafter). The detailed composition of the diets is provided in Table S1. Sturgeon can be distinguished as male or female with the naked eye at the age of 3 years old. Usually, female sturgeons are kept for breeding or caviar, while most male sturgeons are sold for meat. So, in this study, 3-year-old male sturgeons of AS, SS and HS were specifically selected for the purpose of evaluating meat quality.

**Figure 1:**
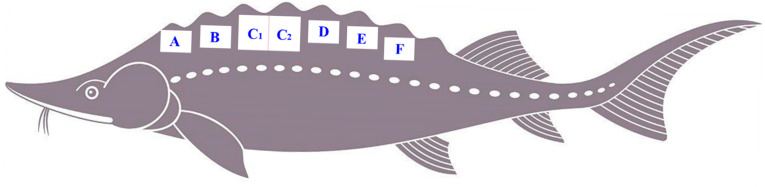
The sampling schematic diagram. A: Metabolomics samples, B: Histological analysis samples, C: Texture analysis samples (C_1_: shear force, C_2_: other textural indices), D: Drip loss samples, E: pH samples, F: Proximate composition samples.

From each experimental group (AS, SS, HS), eight individuals (mean weights: 3.2 ± 0.18, 2.5 ± 0.14, and 3.5 ± 0.21 kg, respectively) were randomly selected. The fish were anesthetized in MS-222 (100 mg/L) and subsequently euthanized. Following skin removal, muscle color parameters (L*, a*, b*) were immediately recorded. A standardized sampling protocol (Figure 1) was then implemented on one side of the dorsal muscle. For this purpose, multiple adjacent samples were collected: 50 mg for metabolomics (snap-frozen in liquid nitrogen), a 0.5 cm^3^ cube for histological fixation in 4% paraformaldehyde, two 2 cm × 1 cm × 1 cm portions for texture profile and shear force analysis, a 20 g block (5 cm × 2 cm × 2 cm) for drip loss determination, a 10 g sample for pH measurement, and finally, a 100 g portion was stored at −20 °C for subsequent analysis of proximate composition.

### 2.2. Proximate Analysis

The contents of crude protein, crude lipid, moisture, and ash were determined as follows: crude protein was quantified via the Kjeldahl method (Foss, Nordborg, Denmark); crude lipid was measured by Soxhlet extraction (Foss, Nordborg, Denmark); moisture content was assessed by drying samples at 105 °C until constant mass was achieved; and ash content was determined after incineration at 550 °C for 16 h using a muffle furnace (CWF1100, Derby, UK) [19].

### 2.3. Amino Acids Analysis

The amino acid composition of filet samples was analyzed with an automatic amino acid analyzer (Hitachi, Ltd., Tokyo, Japan, model 835–50) following acid hydrolysis in 6 M HCl at 110 °C for 24 h. To quantify sulfur-containing amino acids, performic acid oxidation was performed by treating samples with 2 mL of performic acid at 55 °C for 15 min prior to separation on a sodium-type ion-exchange column.

For free amino acid analysis, fresh filet tissue (0.3 g) was homogenized in 9 mL of 5% trichloroacetic acid for 1 min. The homogenate was held at 4 °C for 2 h and subsequently centrifuged at 10,000 rpm for 10 min at 4 °C. The resulting supernatant was adjusted to pH 2 using 6 mol/L NaOH and brought to a final volume of 10 mL. Aliquots of 1 mL were filtered through a 0.22 μm membrane and analyzed using an automated amino acid analyzer (Hitachi High-Tech Corporation, Tokyo, Japan, LA8080 Series) [20].

### 2.4. Fatty Acids Analysis

Methylation of fatty acids from flesh was carried out using BF_3_-methanol prior to GC–MS analysis. Specifically, 2 mL of 14% BF_3_-MeOH was added to the extracted lipid, and the mixture was incubated at 100 °C for 25 min. Then, 2 mL of benzene and 2 mL of methanol were introduced, and heating continued for another 25 min at 100 °C. After cooling, the fatty acid methyl esters were extracted into n-hexane following the addition of distilled water. The mixture was centrifuged at 3000 rpm for 10 min, and the upper n-hexane layer was analyzed by GC–MS (7980B, Agilent Technologies, Inc., Santa Clara, CA, USA) [21].

### 2.5. Muscle Color Analysis

Muscle color measurements were taken on the dorsal area above the lateral line using a calibrated portable Chroma Meter (CM-600d, Konica Minolta, Tokyo, Japan). The instrument recorded the three-dimensional CIELAB color coordinates: L* (lightness), a* (red-green component), and b* (yellow-blue component). In this system, L* scales from black (low values) to white (high values). The chromaticity coordinate a* varies from green (negative values) to red (positive values), and b* varies from blue (negative values) to yellow (positive values) [22].

### 2.6. Drip Loss and pH

Drip loss was determined following the method of Tian et al. [23] with minor adjustments. Muscle specimens (5 cm × 2 cm × 2 cm) were first weighed (D1) and hung inside a 250 mL paper cup. The cup was sealed with cling film and kept at 4 °C for 24 h. Following this, the samples were blotted dry and reweighed (D2). The percentage drip loss was calculated using the formula: 100 × (D1 − D2)/D1.

Meat pH was measured with a specialized pH meter (TESTO-205, Titisee-Neustadt, Germany). The probe was inserted into the muscle, and the value was recorded upon stabilization.

### 2.7. Texture Analysis

A texture analyzer (TMS-PRO, FTC, Sterling, VA, USA) was employed to determine key texture indices such as hardness, chewiness, and shear force, following a published method [10] with modifications. The texture profile analysis was performed using a 37.5 mm radius cylindrical probe, wherein the settings included a test speed of 2.00 mm/s and a deformation of 50% strain. Shear force measurement was carried out with a 0.50 mm thick cutter probe at a test speed of 1.00 mm/s, cutting to 50% of the sample thickness.

### 2.8. Histological Analysis

Muscle samples were prepared for histological examination by hematoxylin and eosin (H&E) staining according to Yu et al. [24]. The stained sections were imaged with an inverted microscope (Nikon CI-S, Tokyo, Japan) and an associated imaging system (Nikon FI2, Japan). Morphometric parameters, including muscle fiber diameter and density, were then quantified from the images using ImageJ software (version 1.53, National Institutes of health, Bethesda, MD, USA), as per the method of Ma et al. [25]. Specifically, three fish (n = 3) for each group were used. From each fish, three non-consecutive sections were taken, and five random field measurements per section were analyzed for histological quantification.

### 2.9. Metabonomics Analysis

Flesh metabolomics was performed on eight individual sturgeon from the AS, SS, and HS groups. Each sample (50 mg) was extracted with 1 mL of cold methanol/acetonitrile/water (2:2:1, *v*/*v*/*v*) via vigorous vortexing. LC-MS/MS analysis was conducted on an Agilent 1290 UHPLC (Agilent Technologies, Inc., Santa Clara, CA, USA) system equipped with a Triple TOF 6600 mass spectrometer (Sciex, Framingham, MA, USA). Quality control (QC) samples, where all samples were equally pooled, were prepared to monitor instrument stability and data reliability. Experimental procedures, including sample preparation, LC-MS/MS analysis, and data preprocessing, were supported by Shanghai Applied Protein Technology Co., Ltd. (Shanghai, China).

Raw data were processed using XCMS software (version 3.8.0) for peak picking, alignment, and integration. Total peak area normalization was applied to correct for overall signal intensity variations among samples. The normalized data were then imported into SIMCA-P 16.1 software (Umetrics, Umea, Sweden) for multivariate statistical analysis.

Differential metabolites were identified based on the following two criteria: (1) variable importance in projection (VIP) > 1.0 from the orthogonal partial least squares-discriminant analysis (OPLS-DA) model, and (2) Student’s *t*-test *p*-value < 0.05 between compared groups. Only metabolites with confident annotations against the HMDB and MetLin databases were retained for further analysis.

Enrichment analysis of KEGG pathways was performed on the identified differential metabolites using the bioinformatics cloud platform (https://www.aptbiotech.com/). Pathways with a *p*-value < 0.05 were considered statistically significant.

### 2.10. Statistical Analysis

Data are presented as mean ± SEM. Normality was assessed using the Shapiro–Wilk test, and homogeneity of variances was evaluated using Levene’s test. All statistical analyses were performed using SPSS for Windows (version 23.0, SPSS Inc., Chicago, IL, USA). Subsequently, one-way ANOVA was employed, and when significant differences were detected, Tukey’s HSD test was used for post hoc multiple comparisons. A significance level of *p* < 0.05 was adopted for all tests.

## 3. Results

### 3.1. Proximate Composition

As shown in Table 1, no significant differences were found among the three sturgeon groups for moisture, crude lipid, crude protein, and ash content (*p* > 0.05). Numerically, HS had slightly higher protein (18.5%) and ash (1.20%), but these differences were not statistically significant. On the whole, the three species exhibited similar proximate compositions.

### 3.2. Amino Acids and Fatty Acids

Comprehensive profiling of amino acids in the muscle of the three sturgeon groups is presented in Table 2. Hydrolyzed amino acid analysis identified 16 amino acids, comprising 8 essential (EAA) and 8 non-essential (NEAA) amino acids, with no significant differences observed across the groups.

In contrast, several free amino acids showed significant differences, especially between AS and HS. Valine (Val) was significantly higher in AS than in SS and HS (*p* < 0.05). Alanine (Ala) and isoleucine (Ile) were also significantly elevated in AS compared to HS (*p* < 0.05), with SS showing an intermediate level. Total free amino acids (TFAA) were significantly higher in AS than in both SS and HS (*p* = 0.010), whereas total delicious amino acids (DAA) showed a similar trend but did not reach significance (*p* = 0.054). No significant differences were detected for other free amino acids (*p* > 0.05). Taken together, HS exhibited a free amino acid profile that more closely resembled that of SS than that of AS.

The fatty acid composition of sturgeon muscle is detailed in Table 3. The profile was predominated by polyunsaturated fatty acids (PUFAs, 43–47%), followed by monounsaturated (MUFAs, 31–35%) and saturated fatty acids (SFAs, 20–21%). Among SFAs, C16:0 was the predominant component, with no significant difference among groups. However, significant differences were observed for several minor SFAs. Specifically, C14:0 was significantly lower in HS than in AS and SS (*p* < 0.05). C18:0 was highest in HS and significantly different from SS (*p* < 0.05). C20:0 was higher in SS than in AS, and undetectable in HS (*p* < 0.05). C22:0 followed a decreasing order of AS > SS > HS (*p* < 0.05). Total SFA content did not differ among groups (*p* > 0.05).

For MUFAs, C18:1N9C was the dominant fatty acid. C16:1N7 was significantly higher in SS than in HS (*p* < 0.05). C24:1N9 was highest in SS, with AS and HS showing similarly lower levels (*p* < 0.05). Notably, C18:1N9T (trans-oleic acid) was lower in HS than in AS and SS, with a borderline significant difference (*p* = 0.050). Total MUFA showed a trend toward higher values in SS but did not reach significance (*p* = 0.058).

Regarding PUFAs, total PUFA content differed significantly among groups (*p* < 0.05), with HS having the highest and SS the lowest values. Specifically, arachidonic acid (C20:4N6, ARA) was significantly higher in HS than in SS (*p* < 0.05), with AS showing an intermediate level. C20:3N6 was significantly higher in AS and HS than in SS (*p* < 0.05). C22:2N6 was significantly lower in both SS and HS compared to AS (*p* < 0.05). C18:3N3 (α-linolenic acid) was slightly lower in HS and SS compared to AS, with a borderline difference (*p* = 0.050). No significant differences were detected for other PUFAs (*p* > 0.05). Collectively, the fatty acid composition of HS matched AS more closely and deviated more from SS.

### 3.3. Flesh Color

The SS and HS groups exhibited significantly higher a* value in the filet compared to the AS group (*p* < 0.05), as summarized in Table 4. For b* value, AS showed significantly lower yellowness than SS (*p* < 0.05), with HS displaying an intermediate value that did not differ significantly from either AS or SS. No significant difference was observed in lightness (L*) among the three groups. Overall, the flesh color of HS and SS was more similar to each other than to that of AS.

### 3.4. Texture, Drip Loss and pH

The texture parameters, drip loss, and pH values of the three sturgeon species are presented in Table 5. HS showed significantly higher hardness and springiness than SS (*p* < 0.05), with AS displaying an intermediate level that did not differ significantly from either HS or SS. Notably, both gumminess and chewiness were significantly higher in HS than in both AS and SS (*p* < 0.05), whereas no significant difference was observed between the two parent species. Shearing force, drip loss, and pH did not differ significantly among the three groups (*p* > 0.05). Thus, HS exhibited superior textural properties, particularly in gumminess and chewiness, compared to both parent species.

### 3.5. Flesh Histology

**Figure 2:**
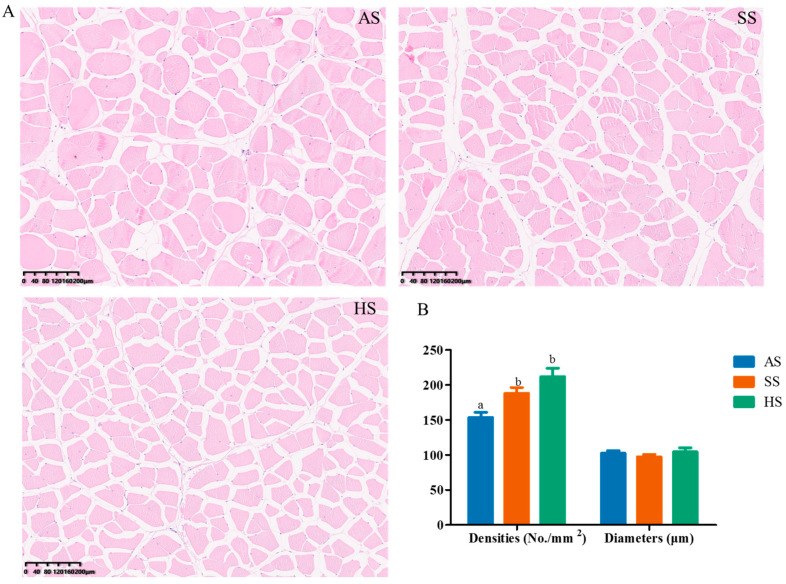
Muscle histology structure in three species of sturgeon. (**A**), histological structure (bar = 200 μm); (**B**), muscle fiber densities (No./mm^2^) and diameters (μm). Values are means ± SEM (*n* = 3 fish per group). For histological quantification, three non-consecutive tissue sections were prepared from each individual fish, and five randomly selected microscopic fields were measured and analyzed per section. Bars with different letters denote significant differences among treatments (*p* < 0.05).

Histological analysis revealed that the muscle fiber density was significantly higher in the SS and HS groups than in the AS group (*p* < 0.05), whereas the muscle fiber diameter showed no significant inter-group differences (Figure 2).

### 3.6. Flesh Metabolite Profiles

#### 3.6.1. Identification and Multivariate Analysis of Metabolites

After data preprocessing, 961 and 622 effective peaks were identified and quantified in the positive and negative ion modes of the muscle samples, respectively. PCA analysis was employed to detect the similarities and differences in the samples. And the reliability of the collected data was monitored using QC sample monitoring equipment. In both positive and negative ion modes, the QC samples are closely distributed near the center point, indicating that the errors caused by the instrument during sample extraction and data acquisition throughout the entire testing process are relatively small, and the test data are reliable (Figure S1A). In order to optimize the separation between groups, OPLS-DA was used to further analyze the muscle samples between groups of different kinds of sturgeon after removing QC samples. As shown in Figure S1B, the samples in three groups showed a trend of intra-group aggregation and inter-group separation in the positive and negative ion modes. The analysis results showed that there were differences in the phenotypes of muscle metabolites in sturgeon of different varieties. Permutation tests validated the PLS-DA models. In positive ion mode, the R^2^ (Q^2^) values for AS vs. SS, AS vs. HS, and SS vs. HS were 0.9582 (−0.1328), 0.9703 (−0.168), and 0.8846 (−0.1807), respectively. In negative ion mode, the values were 0.9517 (−0.1607), 0.9716 (−0.1842), and 0.8751 (0.1416), respectively (Figure S1C). R2 and Q2 of the random model gradually decreased, indicating that the model was robust and free from overfitting. The separation of metabolites between groups was statistically significant and suitable for the following analysis and verification.

#### 3.6.2. Screening and Analysis of Differential Metabolites

**Figure 3:**
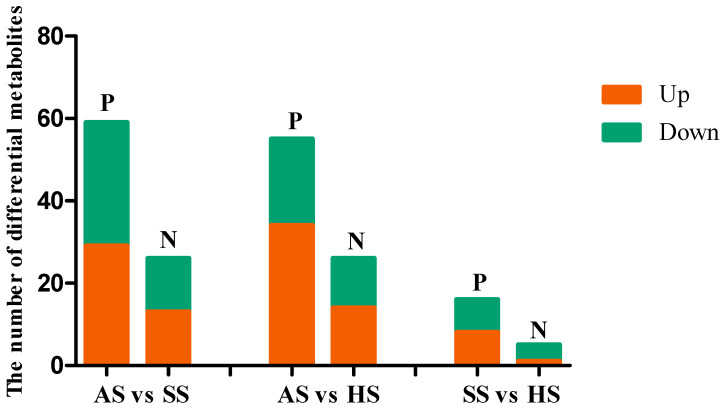
The number of differential metabolites (the screening criteria, VIP > 1.0 and *p* < 0.05) in muscle among three species of sturgeon. P and N, respectively, represent positive and negative ion modes. Red and green indicated significantly up-regulated and down-regulated metabolites, respectively.

Under positive and negative ion modes, differential metabolites were identified by matching the database and applying the screening criteria of VIP > 1.0, *p* < 0.05. In positive ion mode, 59, 55, and 16 differential metabolites were detected in the AS vs. SS, AS vs. HS, and SS vs. HS comparisons, respectively. Among these, 29, 34, and 8 metabolites were upregulated, and 30, 21, and 8 were downregulated in the three comparison groups (Figure 3 and Tables S2–S4). Under negative ion mode, 26, 26, and 5 differential metabolites were identified in the AS vs. SS, AS vs. HS, and SS vs. HS groups, respectively, with 13, 14, and 1 metabolites upregulated, and 13, 12, and 4 downregulated (Figure 3 and Tables S5–S7).

**Figure 4:**
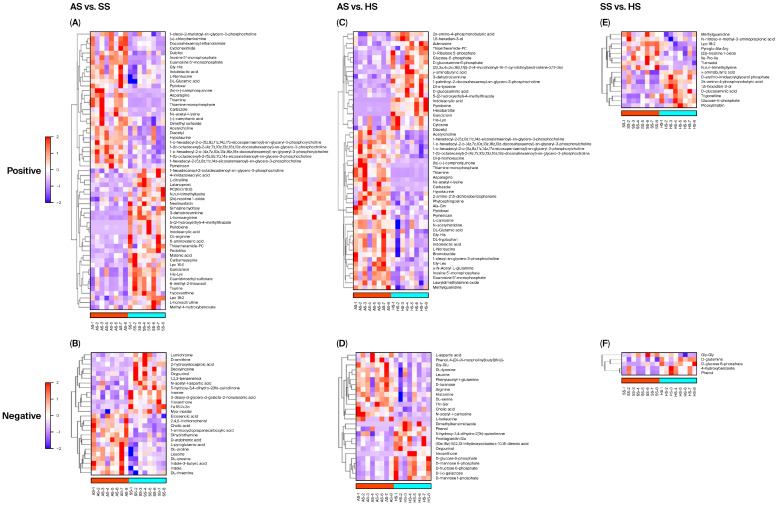
The hierarchical clustering analysis of differential metabolites (the screening criteria, VIP > 1.0 and *p* < 0.05) in muscle among three species of sturgeon. (**A**–**F**) showed the heatmap of hierarchical clustering analysis for AS vs. SS, AS vs. HS, and SS vs. HS in the positive and negative ion modes, respectively. Each row represents a metabolite, and each column represents a sample. Color intensity indicates the relative expression level of each metabolite, with red representing high expression and blue representing low expression. The dendrogram on the left shows hierarchical clustering of metabolites based on similar expression patterns.

Hierarchical clustering analysis showed the significant variations in metabolite profiles between AS vs. SS, AS vs. HS, and SS vs. HS in the positive and negative ion modes, respectively. The differential metabolites with large fold changes were selected and are shown in Table 6 to reveal the difference among the three species of sturgeon, which further corroborates the results presented by the hierarchical clustering analysis. Compared with the SS group, metabolites, such as thiamine, thiamine monophosphate, asparagine, inosine 5′-monophosphate, N.epsilon-acetyl-l-lysine, guanosine 5′-monophosphate, etc., displayed high expressions, whereas D-ornithine, DL-arginine, L-citrulline, L-homoarginine, N,n,n-trimethyllysine, pyridoxine, indoleacrylic acid, etc., revealed low expressions in the muscle of the AS group in two ion modes (Figure 4A,B and Table 6). Nepsilon-acetyl-l-lysine, Dl-beta-homoleucine, asparagine, Gly-Leu, Thr-Ser, L-isoleucine, L-aspartic acid, N-acetyl-l-carnosine, etc., have higher levels in the AS group than those in the HS group, whereas the trends of indoleacrylic acid, gamma.-aminobutyric acid, 2s-amino-4-phosphonobutyric acid, D-glucosaminic acid, Dl-o-tyrosine, D-glucose 6-phosphate, etc., levels were just the opposite (Figure 4C,D and Table 6). Compared with the HS group, the levels of n,n,n-trimethyllysine, Ile-Pro-Ile, Gly-Gly, pyroglu-Ala-Arg, etc., were significantly up-regulated in the SS group. And D-glutamine, D-glucose 6-phosphate, 2s-amino-4-phosphonobutyric acid, gamma.-aminobutyric acid, etc., were obviously down-regulated (Figure 4E,F and Table 6).

Importantly, four differential metabolites were shared between the AS vs. HS and SS vs. HS comparisons, all of which were upregulated in the hybrid sturgeon (HS) compared to both parent species. These common metabolites were γ-Aminobutyric acid (GABA), 2s-Amino-4-phosphonobutyric acid (AP4, D-Glucosaminic acid and D-Glucose 6-phosphate. Thus, HS consistently exhibited higher levels of these four metabolites than both AS and SS, suggesting that these metabolic alterations may contribute to the distinct meat quality characteristics of the hybrid sturgeon.

**Figure 5:**
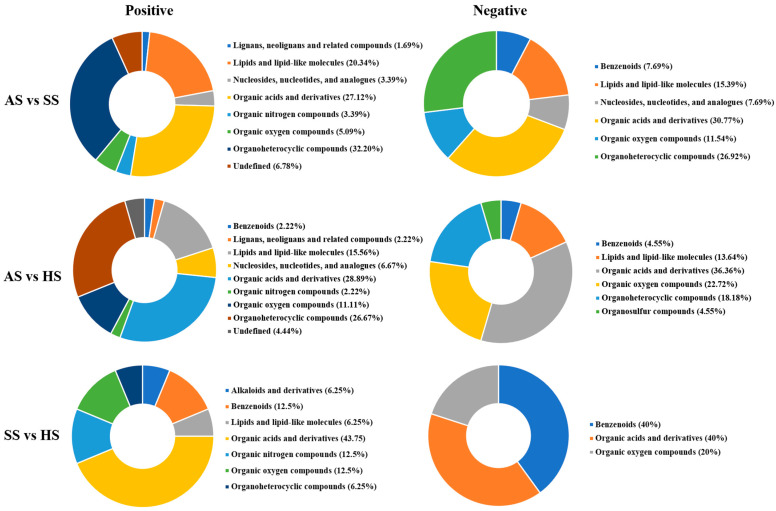
Classification ring diagram of differential metabolites (the screening criteria, VIP > 1.0 and *p* < 0.05) in muscle among AS vs. SS, AS vs. HS, and SS vs. HS in human metabolome database (HMDB). Different colors represent different metabolite classifications.

These differential metabolites were further classified by the HMDB annotation. As shown in Figure 5, the differential metabolites in muscle among three species of sturgeon fell into 10 distinct categories in two modes, which included organic acids and derivatives, lipids and lipid-like molecules, organoheterocyclic compounds, nucleosides, nucleotides and analogs, organic nitrogen compounds, organic oxygen compounds, and so on. Among them, the most metabolites were amino acids and their metabolites.

#### 3.6.3. KEGG Annotation and Enrichment Analysis of Differential Metabolites

**Figure 6:**
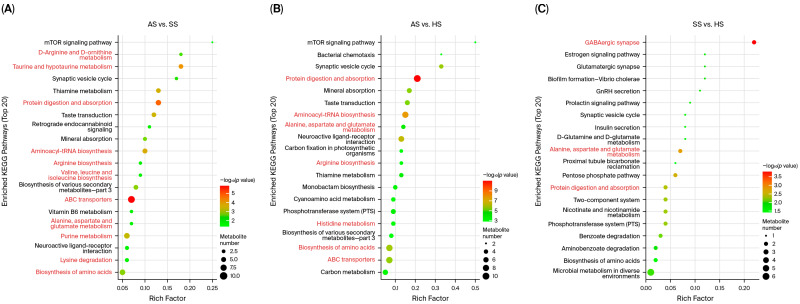
Bubble diagram of KEGG pathways enrichment analysis of significantly different metabolites (top 20) in muscle among three species of sturgeon. (**A**–**C**) showed the bubble diagram of KEGG pathways enrichment analysis for AS vs. SS, AS vs. HS, and SS vs. HS. The Y-axis lists the pathway names, and the X-axis represents the enrichment factor (Rich Factor), calculated as the ratio of differential metabolites annotated to a pathway to the total number of metabolites in that pathway. The size of each bubble is proportional to the number of differential metabolites mapped to the pathway. Bubble color represents the statistical significance of enrichment, with darker red/lower *p*-value indicating more significant enrichment.

KEGG annotation of the differential metabolites revealed their predominant involvement in pathways related to membrane transport, digestive system, amino acid metabolism, nucleotide metabolism, and energy metabolism across three comparative groups. Enrichment analysis further identified the top 20 most significant pathways. In the AS vs. SS comparison, pathways were predominantly enriched in amino acid metabolism (e.g., Taurine and hypotaurine metabolism, Biosynthesis of amino acids, D-Arginine and D-ornithine metabolism, Lysine degradation, Valine, Leucine and isoleucine biosynthesis, Arginine biosynthesis, Alanine, Aspartate and Glutamate metabolism, etc.) alongside ABC transporters, Aminoacyl-tRNA biosynthesis, protein digestion and absorption (Figure 6A). A highly similar enrichment pattern was observed for AS vs. HS (Figure 6B), whereas the SS vs. HS comparison featured pathways like amino acid metabolism, protein digestion and absorption, GABAergic synapse, etc. (Figure 6C).

**Figure 7:**
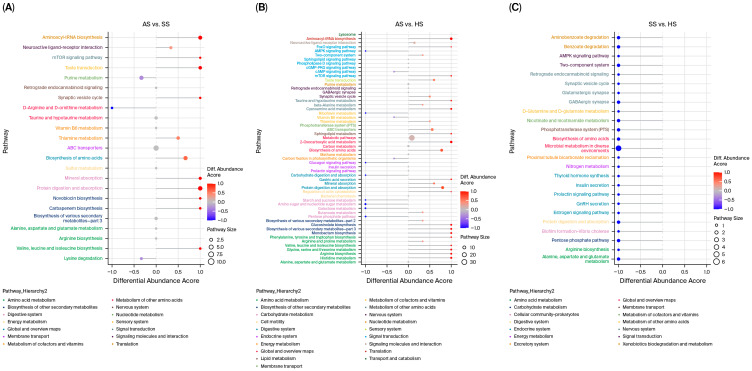
Score plot of KEGG pathways enrichment differential abundance (DA) of different metabolites (the screening criteria, VIP > 1.0 and *p* < 0.05) in muscle among three species of sturgeon. (**A**–**C**) showed the differential abundance score of KEGG pathways enrichment for AS vs. SS, AS vs. HS, and SS vs. HS. The Y-axis lists the pathway names, and the X-axis shows the DA score (ranging from −1 to 1), where positive and negative values indicate overall upregulation and downregulation, respectively, of all identified metabolites in the pathway. The line segment length represents the absolute DA score. The dot size at the end of each line is proportional to the number of metabolites in that pathway. Color intensity (red to blue) reflects the DA score: darker red indicates stronger upregulation, and darker blue indicates stronger downregulation.

The overall changes in differential metabolites in the metabolic pathways enriched by KEGG were further analyzed using the differential abundance score. Taking the most abundant signaling pathway, amino acid metabolism, as an example, valine, leucine and isoleucine biosynthesis were significantly upregulated, while D-arginine and D-ornithine metabolism were significantly downregulated in AS vs. SS (Figure 7A). Compared with the HS group, the muscles of sturgeon in the AS group had significantly up-regulated histidine metabolism, arginine biosynthesis, glycine, serine and threonine metabolism, valine, leucine and isoleucine biosynthesis, phenylalanine, tyrosine and tryptophan biosynthesis, thereby affecting amino acid metabolism (Figure 7B). The differential abundance score of KEGG pathways enrichment was all −1 in SS vs. HS, which indicated that different metabolites of KEGG pathways enrichment were obviously downregulated in the SS group when compared with the HS group. Certainly, amino acid metabolism, involved Alanine, aspartate and glutamate metabolism, Arginine biosynthesis, D-Glutamine and D-glutamate metabolism, were also downregulated (Figure 7C).

## 4. Discussion

As consumers’ demand for high-quality aquatic products continues to rise, understanding the differences in meat quality among various sturgeon species can help farmers produce high-quality sturgeon products that better meet consumers’ preferences, thereby enhancing market competitiveness. The present study conducted a systematic comparative analysis of muscle quality between the hybrid sturgeon and its parental varieties.

In terms of nutritional composition, the hybrid sturgeon has similar conventional nutritional components to its parental varieties, which indicates that these basic nutritional components are relatively stable in the hybrid. Notably, although no statistically significant differences were observed in proximate compositions among the three groups, the crude protein content of hybrid sturgeon reached 18.5%. This value exceeds the protein content of common freshwater species, including grass carp (16.56%), common carp (15.74%), and mandarin fish (16.75%) [26,27,28]. The crude protein content is an important indicator for assessing the nutritional value of fish [17]; the higher protein content of hybrid sturgeon suggests that it has nutritional advantages.

Amino acids serve not only as essential flavor components but also as fundamental biomolecules that play critical roles in various physiological and biochemical processes [29,30]. These versatile compounds participate in numerous vital functions, including cellular signaling, osmoregulation, protein biosynthesis, and metabolic energy provision [31]. In this study, no significant differences were found in the hydrolyzed amino acid profiles of flesh among the three groups, which implied that the composition and content of protein in the muscle of the hybrid sturgeon were similar to those of its parental varieties. This result is consistent with that discovered by Wang et al. [32], who reported that the amino acid content of the hybrid sturgeon (*A*. *baerii* Brandt ♀ × *A*. *schrenckii* Brandt ♂) was similar to that of purebred Siberian sturgeon and Amur sturgeon. Free amino acids are used as an indicator for evaluating the flavor quality of flesh in fish [33,34]. Of these, Glu and Asp are primarily responsible for umami taste; Thr, Gly, Ser, Ala, Lys, and Pro contribute mainly sweetness; while Leu, Val, Iso, His, Phe, Met and Arg predominantly impart bitter notes [35]. According to the results of this study, Glu is the most abundant free amino acid in the muscles of the three sturgeon species examined. In addition to enriching the flavor profile of food [36,37], it also plays a role in regulating various physiological processes, including nervous system function and liver protection [38]. Overall, free amino acids in the muscle of the hybrid sturgeon are closer to those of its maternal variety, the Siberian sturgeon. However, compared with its paternal variety, the Amur sturgeon, both the sweetness (Ala) and bitterness (Val) in the muscle of the hybrid sturgeon decreased. Previous studies indicated that all free amino acids contribute to the present taste of meat [39,40]. No significant difference was observed in the ratio of DAA to TFAA among the muscles of the three sturgeon species, further suggesting that hybridization does not substantially alter muscle nutrition and flavor between the hybrid sturgeon and its parent species. Future studies should incorporate complementary approaches, such as electronic tongue analysis and expression profiling of taste-related genes, to better elucidate the present findings.

Fatty acids serve as fundamental components for cellular structure and play indispensable roles in critical metabolic functions [41]. Fish are widely recognized as a highly nutritious food source, particularly due to their rich content of essential fatty acids, such as PUFAs, in their filets, which confer significant health benefits to humans [42,43]. So, muscle fatty acid contents and composition have always been important indicators for evaluating the muscle quality [44]. In this study, hybrid sturgeons exhibited superior muscle quality characteristics, especially when compared with their paternal variety, Siberian sturgeons. This claim is attributed to the fact that hybrid sturgeons have the lowest content of C18:1N9T (trans-oleic acid) and the highest contents of ΣPUFA and ARA in their muscles. PUFAs can offer dual benefits of flavor enhancement and cardiovascular protection [41,45]. Scientific evidence confirms their ability to reduce cardiovascular disease risk and lower plasma cholesterol levels in humans [46]. ARA can promote brain development, act as a second messenger, and enhance the antioxidant stress resistance and immune capacity of the body [47]. The negative effects of C18:1N9T, as a typical industrial trans-fatty acid, on cardiovascular, metabolic and inflammatory systems have been widely recognized [48]. Overall, fatty acids in the muscles of the hybrid sturgeon, especially the content and level of PUFAs, are closer to those of its parental variety, the Amur sturgeons. Certainly, fish have long been recognized as a vital dietary source of omega-3 fatty acids, particularly due to their high content of EPA and DHA. In nutritional assessments of the flesh of fish, two key fatty acid ratios, PUFA/SFA > 0.4 and n-6/n-3 < 4.0, are commonly employed as evaluation criteria to show the benefits to human health [49,50]. Notably, both hybrid sturgeon and its parental varieties demonstrated favorable values for these ratios in this study, clearly indicating that sturgeon muscle possesses good nutritional quality.

The color and texture of the flesh are important indicators of the quality of fish meat that can directly affect consumer product acceptability and sensory experience [8,51,52]. In this study, the muscle color of hybrid sturgeon more closely resembles that of its maternal variety, exhibiting higher values of a* and b* compared to its paternal variety. Myoglobin has been reported as a key substance that affects the redness of muscles [9,53]. Therefore, the higher a* value may be attributed to the different myoglobin content in the muscles. In addition, the b* value (yellowness) is proven to be strongly associated with lipid oxidation in muscle tissue, as these oxidative processes can generate yellow compounds [54]. Corresponding to the higher b* value, muscle composition analysis revealed relatively higher crude fat content in SS and HS compared to AS, suggesting the possible contribution of lipid oxidation to the muscle color. To better elucidate the mechanisms underlying color variation, future studies should quantify myoglobin content and assess lipid peroxidation levels in muscle tissue.

Fish muscle is the main edible part of the fish consumed by humans [55]. Its quality has always been a critical practical concern for both consumers and producers in the aquaculture industry. In recent years, significant progress has been made in enhancing fish meat quality through texture modification, leading to improved economic outcomes in aquaculture. Notable examples include the development of crispy grass carp [10,25] and crispy tilapia [55,56]. These results all indicate that texture characteristics are important parameters revealing the quality of fish meat [57]. In our study, the enhanced hardness, gumminess and chewiness were observed in the muscle of hybrid sturgeon versus the parent species. Hardness, gumminess and chewiness are the most important criteria for judging the sensory experience of humans on fish meat [58]. And poor hardness will lead to the decline of meat value in secondary processing [59]. The result of texture profile analysis in this study confirmed significant improvements in the muscle of hybrid sturgeon, which provided compelling evidence of heterosis effects on meat quality parameters. Currently, heterosis in meat quality traits has been well-documented in livestock and poultry species [60,61,62], while research on aquatic animals remains limited. This knowledge gap presents novel opportunities for future investigations into improving the meat quality of aquatic species through hybridization strategies.

Fish muscle development occurs through two distinct processes: hyperplasia (increase in muscle fiber number) and hypertrophy (expansion of muscle fiber diameter) [63,64]. These structural modifications directly influence flesh texture and organoleptic quality [8]. Specifically, variations in muscle fiber density and diameter are key determinants of filet texture characteristics. Interestingly, this study revealed that the hybrid sturgeon exhibited the highest number of muscle fiber hyperplasia among the three experimental groups, with a statistically significant increase compared to its paternal variety, the Amur sturgeon. These findings provide a mechanistic explanation for the observed improvements in textural properties, such as gumminess and chewiness, in hybrid sturgeon muscle. Furthermore, this is likely to explain the growth advantage of hybrid sturgeon, which attained the highest body weight among the three species reared under identical conditions from the same batch. So a hypothesis was put forward that hybrid sturgeons enhance muscle texture and growth primarily through hyperplasia (increased muscle fiber density). Nevertheless, further investigation is required to elucidate the underlying mechanisms.

The metabolic profile of meat significantly impacts both human health and flavor development [65]. In this study, we characterized the muscle metabolic profiles of hybrid sturgeon and its parental species, providing the first comparative analysis of their metabolic differences. PCA and OPLS-DA revealed significant metabolic profile differences among the three kinds of sturgeon. Notably, the hybrid exhibited 81 differentially regulated metabolites versus its paternal variety (Amur sturgeon), contrasting with just 21 differences relative to its maternal variety (Siberian sturgeon). The differential metabolites in the hybrid sturgeon and its parental varieties’ muscle included many types, but these are mainly amino acids and their metabolites. The results suggest that there are differences in amino acid metabolism and synthesis between hybrid sturgeons and their parental varieties, which may be an essential reason for the differences in their meat quality. KEGG pathway analysis revealed that the differential metabolites between hybrid sturgeons and their parental varieties were significantly enriched in ABC transporters, protein digestion and absorption and amino acid metabolism, which were the critical processes for amino acid metabolism and synthesis. The ABC transporter family represents one of the largest and most functionally diverse transporter systems in living organisms [66]. These transporters facilitate the active transport of a wide range of substrates, including sugars, amino acids, peptides, proteins, bacterial secretions, metabolites, and oligonucleotides [67]. It is speculated that ABC transporters may influence meat quality regulation through their substrate transport activities, potentially affecting flavor development and texture [68,69]. Through modulating amino acid availability and their subsequent metabolism, the protein digestion-absorption system and amino acid metabolic networks collectively determine muscle growth potential and flavor precursor generation [70]. Interestingly, γ-aminobutyric acid (GABA), 2s-amino-4-phosphonobutyric acid (AP4) and D-glucosaminic acid were obviously up-regulated in hybrid sturgeon muscle compared with the two parental varieties. GABA, a non-protein amino acid synthesized from glutamate by glutamate decarboxylase, serves as the primary inhibitory neurotransmitter in the vertebrate central nervous system and also modulates peripheral muscle tone and contractility [71,72,73,74]. In hybrid sturgeon muscle, the significantly higher level of GABA may be associated with improved texture parameters, particularly greater adhesiveness and chewiness, compared to both parent species. The possible reason is that GABA influences calcium ion (Ca^2+^) homeostasis in muscle fibers by modulating Ca^2+^ release from the sarcoplasmic reticulum, which can delay the onset of post-mortem rigor mortis and reduce muscle shortening [75,76]. If this occurs, such a delay might preserve sarcomere integrity, thereby potentially contributing to the enhanced chewiness observed in hybrid sturgeon. However, direct assessment of calcium dynamics and the stiffening process after death would be required to confirm this possibility. Additionally, the elevated GABA concentration in hybrid sturgeon muscle may also be associated with stress resistance, as previously suggested for other species [77,78,79]. D-Glucosaminic acid is an oxidized derivative of D-glucosamine, which serves as a key precursor for glycosaminoglycans (GAGs, e.g., chondroitin sulfate and hyaluronic acid) [80,81]. GAGs are essential structural components of the extracellular matrix (ECM) and the endomysium surrounding muscle fibers, which can directly affect the hardness and chewiness of muscle [82,83]. So, we hypothesize that the higher level of D-glucosaminic acid in hybrid sturgeon muscle could reflect increased GAG turnover, which in turn might affect muscle chewiness. 2s-Amino-4-phosphonobutyric acid (AP4) is a structural analog of glutamate and a potent agonist at group III metabotropic glutamate receptors (mGluRs) [84]. Beyond its neuroactive properties, AP4 and related phosphoamino acids are involved in phospholipid metabolism and cellular signaling pathways that regulate protein synthesis and degradation [85,86]. It is possible that the elevated AP4 level in hybrid sturgeon muscle is related to the observed differences in free amino acid profile (lower Val, Ile, and Ala than in AS) as well as the enrichment of ABC transporters and amino acid metabolism pathways. On the whole, the current results suggest that amino acid metabolism may underlie the enhanced growth performance, muscle quality and stress resistance observed in hybrid sturgeons. Still, this study has several constraints. The metabolomic findings lack functional validation (e.g., gene expression or enzyme assays), and the sample size per group was limited. Additionally, no sensory analysis or electronic gustatory assessment was performed to corroborate the instrumental results. Future research should address these gaps to confirm the proposed regulatory mechanisms. Additionally, body weight will be included as a covariate, or weight-matched designs will be adopted to further extend our research.

## 5. Conclusions

This study demonstrates that the meat quality of the hybrid sturgeon “Jinglong 1” combines desirable traits from both parent species. The hybrid closely resembles the Siberian sturgeon in most quality attributes, while showing similarity to the Amur sturgeon in polyunsaturated fatty acid composition. Notably, the hybrid exhibits superior textural properties (gumminess and chewiness) compared to both parent species. Metabolomic analysis reveals that these differences are associated with key metabolic pathways, including ABC transporters, protein digestion and absorption, and amino acid metabolism, with several differential metabolites identified as potential regulators. These findings provide a scientific basis for further breeding and commercial development of “Jinglong 1” as a high-quality aquaculture variety.

## Figures and Tables

**Table 1:** Flesh proximate composition in three species of sturgeon (g/100 g).

	AS	SS	HS	*p*-Values
Moisture	74.1 ± 0.77	72.35 ± 1.82	72.95 ± 0.49	0.196
Crude lipid	5.13 ± 0.33	7.20 ± 0.60	6.97 ± 0.75	0.132
Crude protein	18.33 ± 0.53	17.05 ± 0.80	18.5 ± 0.47	0.246
Ash	1.08 ± 0.05	1.08 ± 0.06	1.20 ± 0.00	0.131

Note: Values are presented as mean ± SEM (n = 8 per treatment).

**Table 2:** Flesh amino acid compositions in three species of sturgeon.

	AS	SS	HS	*p*-Values
Hydrolyzed amino acids (g/100 g)				
Lysine (Lys) #	1.70 ± 0.088	1.65 ± 0.11	1.79 ± 0.038	0.515
Methionine (Met) #	0.57 ± 0.035	0.56 ± 0.032	0.61 ± 0.016	0.474
Valine (Val) #	0.78 ± 0.038	0.79 ± 0.047	0.87 ± 0.021	0.211
Threonine (Thr) #	0.78 ± 0.039	0.76 ± 0.052	0.84 ± 0.017	0.324
Phenylalanine (Phe) #	0.80 ± 0.027	0.76 ± 0.053	0.85 ± 0.015	0.282
Leucine (Leu) #	1.45 ± 0.070	1.40 ± 0.085	1.53 ± 0.025	0.409
Isoleucine (Ile) #	0.74 ± 0.035	0.75 ± 0.041	0.82 ± 0.017	0.186
Arginine (Arg)	1.06 ± 0.053	1.01 ± 0.075	1.12 ± 0.027	0.443
Histidine (His) #	0.60 ± 0.017	0.56 ± 0.058	0.51 ± 0.033	0.320
Alanine (Ala)	1.04 ± 0.046	1.00 ± 0.044	1.05 ± 0.025	0.658
Glycine (Gly)	0.80 ± 0.024	0.73 ± 0.053	0.81 ± 0.038	0.343
Glutamic acid (Glu)	2.95 ± 0.160	2.82 ± 0.11	3.11 ± 0.084	0.361
Tyrosine (Tyr)	0.63 ± 0.028	0.63 ± 0.047	0.68 ± 0.014	0.504
Proline (Pro)	0.51 ± 0.028	0.51 ± 0.032	0.63 ± 0.061	0.123
Serine (Ser)	0.75 ± 0.029	0.72 ± 0.049	0.81 ± 0.018	0.238
Aspartic acid (Asp)	1.85 ± 0.085	1.77 ± 0.109	1.98 ± 0.027	0.231
EAA	7.43 ± 0.34	7.24 ± 0.45	7.84 ± 0.12	0.468
NEAA	9.61 ± 0.44	9.21 ± 0.53	10.19 ± 0.24	0.302
TAA	17.04 ± 0.78	16.45 ± 0.98	18.03 ± 0.31	0.361
EAA/TAA	0.44 ± 0.0018	0.44 ± 0.0029	0.43 ± 0.0052	0.584
Free amino acids (μg/g)				
Methionine (Met)	180.00 ± 23.09	200.00 ± 0.00	130.00 ± 10.00	0.292
Valine (Val)	337.50 ± 22.87 ^a^	223.33 ± 20.28 ^b^	213.33 ± 12.02 ^b^	0.005
Phenylalanine (Phe) *	202.50 ± 17.97	213.33 ± 13.33	170.00 ± 37.86	0.486
Leucine (Leu)	307.50 ± 28.39	233.33 ± 37.12	223.33 ± 24.04	0.153
Isoleucine (Ile)	197.50 ± 11.09 ^a^	143.33 ± 21.86 ^ab^	116.67 ± 3.33 ^b^	0.010
Histidine (His)	215.00 ± 52.36	225.00 ± 45.00	220.00 ± 0.00	0.991
Alanine (Ala) *	605.00 ± 18.48 ^a^	360.00 ± 89.63 ^ab^	353.33 ± 71.72 ^b^	0.028
Glycine (Gly) *	230.00 ± 34.88	163.33 ± 3.33	175.00 ± 15.00	0.256
Glutamic acid (Glu) *	882.50 ± 32.76	650.00 ± 119.30	633.33 ± 116.09	0.133
DAA	1920.00 ± 62.58	1386.67 ± 202.18	1273.33 ± 247.27	0.054
TFAA	3150.25 ± 154.20 ^a^	2273.33 ± 216.97 ^b^	2350.00 ± 90.18 ^b^	0.010
DAA/TFAA	0.61 ± 0.014	0.60 ± 0.032	0.54 ± 0.088	0.519

Note: Values are presented as mean ± SEM (n = 8 per treatment). Within a row, mean values bearing different superscript letters are significantly different (*p* < 0.05) as determined by one-way ANOVA. The abbreviations EAA, NEAA, TAA, DAA, and TFAA refer to total essential amino acids, total non-essential amino acids, total amino acids, total delicious amino acids, and total free amino acids, respectively. The symbols # and * denote EAA and DAA, respectively.

**Table 3:** Flesh fatty acid composition in three species of sturgeon (percentage of identified fatty acids, %).

	AS	SS	HS	*p*-Values
C14:0	1.10 ± 0.00 ^a^	1.11 ± 0.04 ^a^	0.92 ± 0.03 ^b^	0.001
C15:0	0.18 ± 0.00	0.18 ± 0.01	0.09 ± 0.05	0.116
C16:0	16.42 ± 0.2	15.85 ± 0.81	16.09 ± 0.14	0.724
C17:0	0.17 ± 0.06	0.21 ± 0.01	0.11 ± 0.07	0.438
C18:0	3.13 ± 0.13 ^ab^	2.78 ± 0.12 ^b^	3.65 ± 0.22 ^a^	0.013
C20:0	0.03 ± 0.03 ^b^	0.14 ± 0.02 ^a^	0.00 ± 0.00 ^b^	0.001
C21:0	0.05 ± 0.03	0.02 ± 0.02	0.00 ± 0.00	0.245
C22:0	0.25 ± 0.01 ^a^	0.15 ± 0.02 ^b^	0.03 ± 0.03 ^c^	0.000
C24:0	0.00 ± 0.00	0.01 ± 0.01	0.00 ± 0.00	0.405
∑SFA	21.34 ± 0.33	20.45 ± 0.74	20.89 ± 0.11	0.445
C16:1N7	1.85 ± 0.04 ^ab^	2.19 ± 0.14 ^a^	1.74 ± 0.07 ^b^	0.019
C17:1N7	0.00 ± 0.00	0.06 ± 0.02	0.03 ± 0.03	0.118
C18:1N9C	29.09 ± 0.7	31.78 ± 0.89	28.99 ± 1.07	0.094
C18:1N9T	0.20 ± 0.01	0.19 ± 0.05	0.07 ± 0.04	0.050
C20:1	1.38 ± 0.06	1.19 ± 0.13	1.09 ± 0.07	0.117
C22:1N9	0.05 ± 0.03	0.07 ± 0.02	0.02 ± 0.02	0.342
C24:1N9	0.02 ± 0.02 ^b^	0.08 ± 0.02 ^a^	0.00 ± 0.00 ^b^	0.010
∑MUFA	32.59 ± 0.83	35.57 ± 0.99	31.92 ± 1.08	0.058
C18:2N6C	30.60 ± 0.32	31.10 ± 0.99	31.93 ± 0.85	0.500
C18:3N3	2.97 ± 0.07	2.72 ± 0.03	2.66 ± 0.11	0.050
C18:3N6	1.09 ± 0.08	1.41 ± 0.20	1.74 ± 0.34	0.193
C20:2	1.55 ± 0.03	1.42 ± 0.02	1.44 ± 0.11	0.412
C20:3N3	0.29 ± 0.01	0.23 ± 0.02	0.25 ± 0.04	0.257
C20:3N6	0.77 ± 0.04 ^a^	0.52 ± 0.04 ^b^	0.73 ± 0.03 ^a^	0.003
C20:4N6 (ARA)	1.39 ± 0.14 ^ab^	1.03 ± 0.11 ^b^	1.64 ± 0.11 ^a^	0.018
C20:5N3 (EPA)	1.89 ± 0.11	1.48 ± 0.18	1.64 ± 0.09	0.144
C22:2N6	0.32 ± 0.00 ^a^	0.22 ± 0.01 ^b^	0.22 ± 0.01 ^b^	0.000
C22:6N3 (DHA)	5.20 ± 0.42	3.85 ± 0.71	4.95 ± 0.49	0.236
∑n-3 PUFA	10.35 ± 0.56	8.27 ± 0.88	9.50 ± 0.54	0.150
∑n-6 PUFA	33.40 ± 0.34	33.76 ± 0.79	35.52 ± 1.15	0.207
∑PUFA	46.07 ± 0.66 ^ab^	43.98 ± 0.64 ^b^	47.19 ± 0.97 ^a^	0.045
∑UFA	78.66 ± 0.33	79.55 ± 0.74	79.11 ± 0.11	0.445
n-6/n-3	3.33 ± 0.18	4.36 ± 0.74	3.87 ± 0.32	0.313
PUFA/SFA	2.16 ± 0.04	2.16 ± 0.08	2.26 ± 0.03	0.406

Note: Values are presented as mean ± SEM (n = 8 per treatment). Within a row, mean values bearing different superscript letters are significantly different (*p* < 0.05) as determined by one-way ANOVA. SFA, MUFA, PUFA and UFA indicated saturated fatty acids, monounsaturated fatty acids, polyunsaturated fatty acids and unsaturated fatty acids, respectively.

**Table 4:** Flesh color in three species of sturgeon.

	AS	SS	HS	*p*-Values
L*	54.92 ± 1.15	56.01 ± 0.51	54.82 ± 1.39	0.693
a*	−2.69 ± 0.21 ^b^	−1.03 ± 0.43 ^a^	−1.16 ± 0.50 ^a^	0.013
b*	5.88 ± 0.68 ^b^	10.29 ± 0.96 ^a^	9.40 ± 1.27 ^ab^	0.013

Note: Values are presented as mean ± SEM (n = 8 per treatment). Within a row, mean values bearing different superscript letters are significantly different (*p* < 0.05) as determined by one-way ANOVA. L*, the brightness; a*, redness; b*, yellowness.

**Table 5:** Texture profile, drip loss and pH of muscle in three species of sturgeon.

	AS	SS	HS	*p*-Values
Hardness (N)	174.88 ± 16.44 ^ab^	126.40 ± 14.22 ^b^	187.49 ± 12.96 ^a^	0.017
Springiness (mm)	2.79 ± 0.22 ^ab^	2.11 ± 0.12 ^b^	3.11 ± 0.28 ^a^	0.009
Gumminess (N)	52.63 ± 1.58 ^b^	54.48 ± 2.19 ^b^	68.97 ± 3.79 ^a^	0.002
Chewiness (mj)	144.99 ± 13.51 ^b^	124.48 ± 9.54 ^b^	198.51 ± 14.85 ^a^	0.005
Shearing force (N)	186.03 ± 15.48	158.21 ± 18.06	180.61 ± 15.96	0.273
Drip loss	3.09 ± 0.33	3.52 ± 0.51	2.92 ± 0.29	0.562
pH	6.79 ± 0.10	6.88 ± 0.09	6.86 ± 0.10	0.790

Note: Values are presented as mean ± SEM (n = 8 per treatment). Within a row, mean values bearing different superscript letters are significantly different (*p* < 0.05) as determined by one-way ANOVA.

**Table 6:** The differential metabolites with large fold changes (FC) in three species of sturgeon.

No.	AS vs. SS			AS vs. HS			SS vs. HS		
	Name	Log_2_FC	Types	Name	Log_2_FC	Types	Name	Log_2_FC	Types
1	Thiamine	4.02	up	Thiamine	4.64	up	N,n,n-trimethyllysine	1.65	up
2	Thiamine monophosphate	3.54	up	N.epsilon.-acetyl-l-lysine	4.22	up	Ile-Pro-Ile	1.16	up
3	Asparagine	3.51	up	Thiamine monophosphate	4.11	up	Pyroglu-Ala-Arg	0.89	up
4	Inosine 5′-monophosphate	3.08	up	Dl-.beta.-homoleucine	3.22	up	Gly-Gly	0.74	up
5	N.epsilon.-acetyl-l-lysine	2.52	up	Asparagine	2.98	up	Tramadol	0.72	up
6	Guanosine 5′-monophosphate	1.94	up	Gly-Leu	2.86	up	Lpc 18:2	0.70	up
7	D-arabinonic acid	1.51	up	Thr-Ser	2.61	up	(2s)-nicotine 1-oxide	0.41	up
8	Docosahexaenoyl ethanolamide	1.49	up	L-isoleucine	1.91	up	Methylguanidine	0.28	up
9	Hypotaurine	1.43	up	L-aspartic acid	1.91	up	gamma.-aminobutyric acid	−0.69	down
10	5-aminovaleric acid	−1.79	down	N-acetyl-l-carnosine	1.60	up	D-glutamine	−0.89	down
11	D-ornithine	−1.80	down	Hypotaurine	1.57	up	Phenol	−1.17	down
12	DL-arginine	−1.83	down	Inosine 5′-monophosphate	1.56	up	4-hydroxybenzoate	−1.18	down
13	2-hydroxyisocaproic acid	−1.89	down	gamma.-aminobutyric acid	−0.92	down	Trigonelline	−1.28	down
14	L-citrulline	−1.95	down	2s-amino−4-phosphonobutyric acid	−1.06	down	Glucose-6-phosphate	−1.32	down
15	L-homoarginine	−2.03	down	D-glucosaminic acid	−1.79	down	D-erythro-imidazolylglycerol phosphate	−1.36	down
16	3-dehydrocarnitine	−2.62	down	D-glucose 6-phosphate	−2.04	down	2s-amino-4-phosphonobutyric acid	−1.41	down
17	N,n,n-trimethyllysine	−3.02	down	3-dehydrocarnitine	−2.05	down	1,5-hexadien-3-ol	−1.77	down
18	Irisxanthone	−3.05	down	Dl-o-tyrosine	−2.08	down	D-glucose 6-phosphate	−1.81	down
19	Pyridoxine	−3.66	down	Pyridoxine	−4.00	down	Picoxystrobin	−1.83	down
20	Indoleacrylic acid	−8.62	down	Indoleacrylic acid	−8.98	down	D-glucosaminic acid	−1.83	down

## Data Availability

The original contributions presented in this study are included in the article/Supplementary Material. Further inquiries can be directed to the corresponding author.

## Supplementary Materials

The following supporting information can be downloaded at: https://www.mdpi.com/article/10.3390/foods15101665/s1, Figure S1: Multivariate analysis of muscle metabolites; Table S1: Proximate composition of experimental diets; Table S2: Identification of differential metabolites in the flesh of AS vs. SS by LC-MS (positive mode); Table S3: Identification of differential metabolites in the flesh of AS vs. HS by LC-MS (positive mode); Table S4: Identification of differential metabolites in the flesh of SS vs. HS by LC-MS (positive mode); Table S5: Identification of differential metabolites in the flesh of AS vs. SS by LC-MS (negative mode); Table S6: Identification of differential metabolites in the flesh of AS vs. HS by LC-MS (negative mode); Table S7: Identification of differential metabolites in the flesh of SS vs. HS by LC-MS (negative mode).
