# Supplementary material for: A Comparative Analysis of Muscle Nutritional Composition, Texture, Microstructure, and Metabolomics: Hybrid Sturgeon (Acipenser baerii Brandt ♀ × Acipenser schrenckii Brandt ♂) Versus Its Parent Varieties
Source: Foods. 2026 May 10;15(10):1665. doi: 10.3390/foods15101665 (PMC13206672; doi:10.3390/foods15101665)
Supplement: Supplementary file 1 [file foods-15-01665-s001.zip › foods-4274432-supplementary/Supplementary Files/Supplementary material-Figure.pdf]

A comparative analysis of muscle nutritional composition, texture, microstructure, and metabolomics: Hybrid sturgeon (*Acipenser baerii* Brandt ♀ × *Acipenser schrenckii* Brandt ♂) versus its parent varieties

Guanling Xu, Wei Xing, Ying Zhang, Tieliang Li, Tingting Song, Lin Luo, Huanhuan Yu*

Fisheries Research Institute, Beijing Academy of Agriculture and Forestry Sciences, Beijing, 100068, China.

* Corresponding author. E-mail address: yuhuanhuan@baafs.net.cn

Supplementary Material

Figure S1. Multivariate analysis of muscle metabolites.

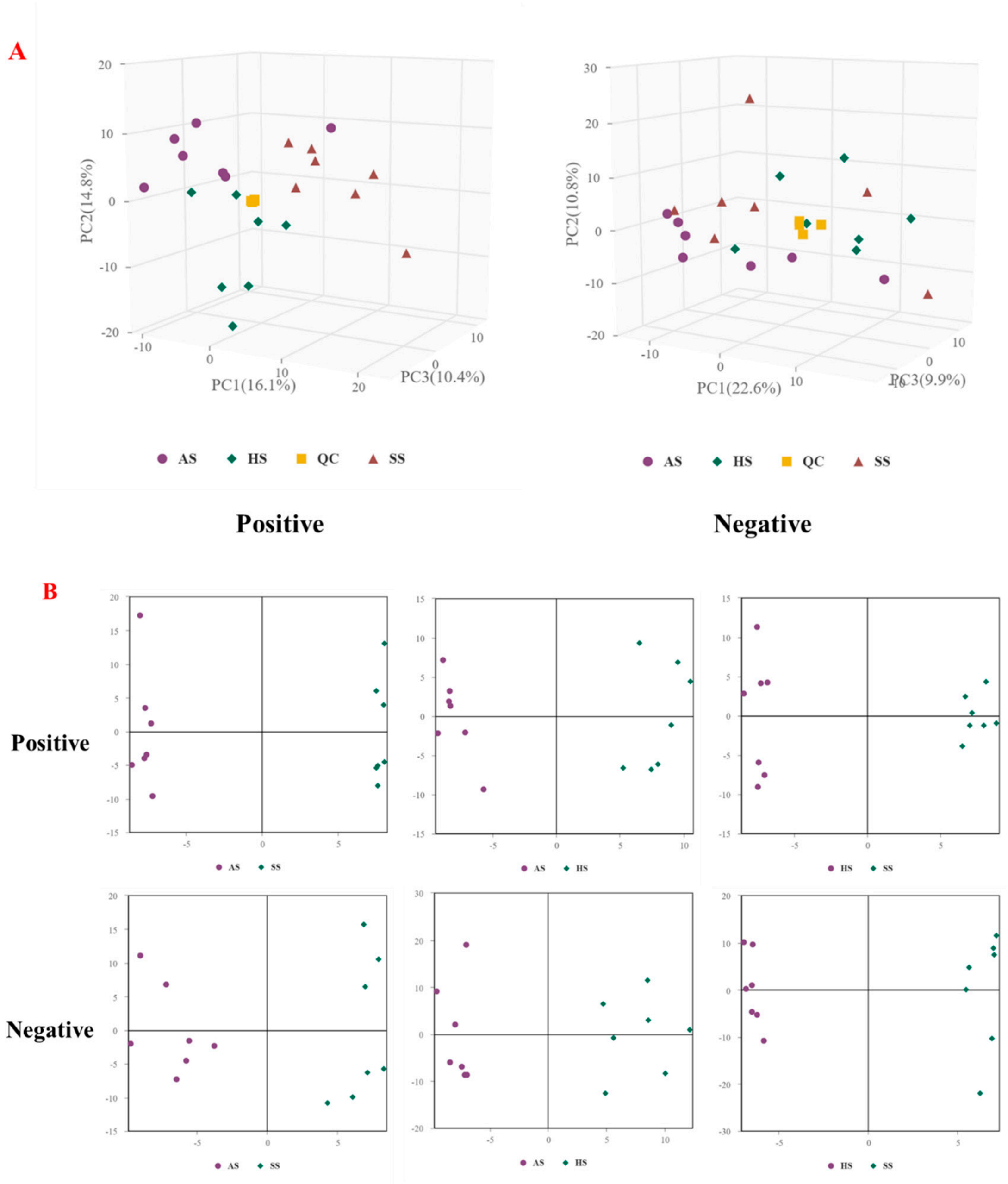

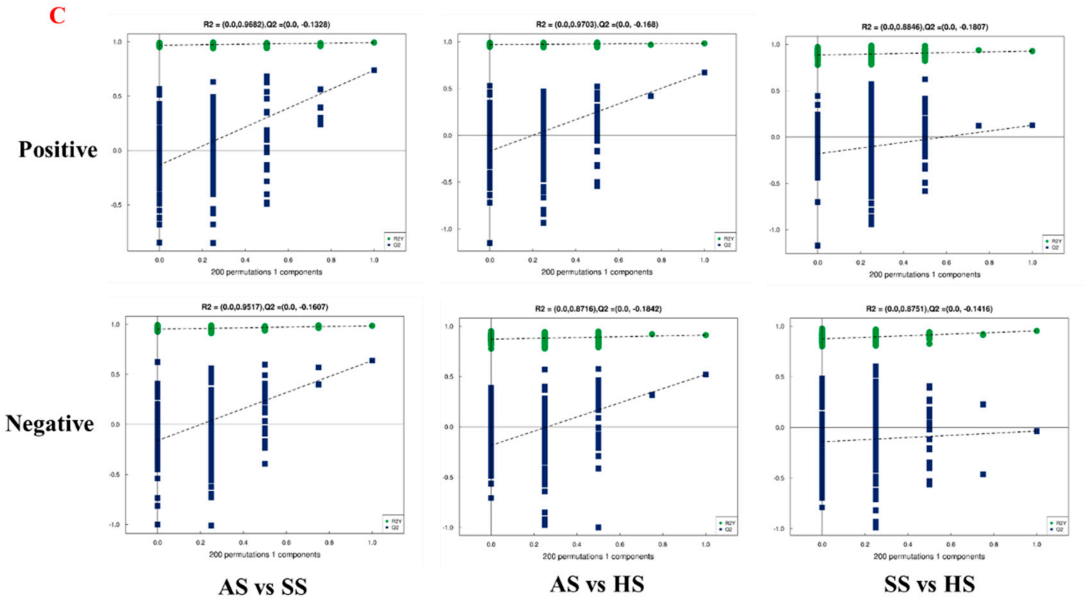

Figure S1. Multivariate analysis of muscle metabolites. A, score scatter plot of PCA model. B, OPLS-DA model for AS vs SS, AS vs HS, and SS vs HS. C, permutation test of OPLS-DA model for AS vs SS, AS vs HS, and SS vs HS.
